# Bycatch in the Maldivian pole-and-line tuna fishery

**DOI:** 10.1371/journal.pone.0177391

**Published:** 2017-05-24

**Authors:** Kelsey I. Miller, Ibrahim Nadheeh, A. Riyaz Jauharee, R. Charles Anderson, M. Shiham Adam

**Affiliations:** 1 International Pole & Line Foundation, London, United Kingdom; 2 Marine Research Centre, Malé, Republic of Maldives; North Carolina State University, UNITED STATES

## Abstract

Tropical tuna fisheries are among the largest worldwide, with some having significant bycatch issues. However, pole-and-line tuna fisheries are widely believed to have low bycatch rates, although these have rarely been quantified. The Maldives has an important pole-and-line fishery, targeting skipjack tuna (*Katsuwonus pelamis)*. In the Maldives, 106 pole-and-line tuna fishing days were observed between August 2014 and November 2015. During 161 fishing events, tuna catches amounted to 147 t: 72% by weight was skipjack, 25% yellowfin tuna (*Thunnus albacares)* and 3% other tunas. Bycatch (all non-tuna species caught plus all tuna discards) amounted to 951 kg (0.65% of total tuna catch). Most of the bycatch (95%) was utilized, and some bycatch was released alive, so dead discards were particularly low (0.02% of total tuna catch, or 22 kg per 100 t). Rainbow runner (*Elagatis bipinnulata*) and dolphinfish (*Coryphaena hippurus*) together constituted 93% of the bycatch. Live releases included small numbers of silky sharks (*Carcharhinus falciformis*) and seabirds (noddies, *Anous tenuirostris* and *A*. *stolidus*). Pole-and-line tuna fishing was conducted on free schools and schools associated with various objects (Maldivian anchored fish aggregating devices [aFADs], drifting FADs from western Indian Ocean purse seine fisheries, other drifting objects and seamounts). Free school catches typically included a high proportion of large skipjack and significantly less bycatch. Associated schools produced more variable tuna catches and higher bycatch rates. Fishing trips in the south had significantly lower bycatch rates than those in the north. This study is the first to quantify bycatch rates in the Maldives pole-and-line tuna fishery and the influence of school association on catch composition. Ratio estimator methods suggest roughly 552.6 t of bycatch and 27.9 t of discards are caught annually in the fishery (based on 2015 national catch), much less than other Indian Ocean tuna fisheries, e.g. gillnet, purse-seine, and longline.

## Introduction

Bycatch in fisheries is a major environmental and resource management problem. Bycatch can be defined in several ways, most often as that part of the catch which is not targetted, but perhaps most meaningfully as that part of the catch which is discarded dead. Minimizing bycatch is one of the most significant challenges facing fisheries today [1] and is of concern for two main reasons. First, there is the issue of waste. A recent global review suggested that discards averaged 10.3 Mt per year during 2000–2010 [2]. While some discards are of non-edible or otherwise non-commercial species, much is of potential value. Indeed, in some fisheries a large proportion of the discards can be of the target species, if they are in poor condition or small-sized. For example, one review noted that over 220 t of (mainly under-sized) tuna were discarded for every 1000 t of tuna landed from purse seine sets on log-schools in the Pacific [3].

Second, there is the issue of ecological impacts, both on individual species and more generally on the wider pelagic ecosystem. There is particular concern for some endangered, threatened and protected (ETP) species, including larger and longer-lived ‘charismatic megafauna’ such as marine mammals, seabirds, marine turtles and sharks [4]. Some impacted species have such low population sizes that even apparently minor bycatch may have grave consequences. Within the Indian Ocean, for example, the survival of the critically endangered Amsterdam Albatross (*Diomedea amsterdamensis*) is threatened by the bycatch of just a handful of individuals each year in tuna longline fisheries [5]. Also within the Indian Ocean, removal of top predators by tuna fisheries is implicated in ecosystem changes marked by the increase in abundance of mid-sized predators including crocodile shark (*Pseudocarcharias kamoharai*) and lancetfish (*Alepisaurus ferox*), with unknown longer-term consequences [6]. In turn, concerns over such conservation and ecosystem issues can have negative impacts on fishers, for example with the imposition of fishery closures or trade embargoes (e.g. [7, 8].

Tunas are among the most sought-after of commercial food fishes, and in the Indian Ocean recent catches of tuna and tuna-like species have been in excess of 1.6 Mt per year. Catches of the three main tropical species amounted to 527,447 t in 2015, of which yellowfin tuna (*Thunnus albacares*) accounted for 45%, followed closely by skipjack tuna (*Katsuwonus pelamis*) (44%), and catches of bigeye tuna (*Thunnus obesus*) account for the remaining 11%. The major tuna fishing gears within the entire Indian Ocean (both tropical and temperate) are purse seine (26% of tuna catch during 2015), gillnet (33%), longline (16%) and pole-and-line (6%), with the balance taken by minor gears or unrecorded gear types (tuna and effort catch data from www.iotc.org, accessed January 2017).

Bycatch issues have not been adequately addressed in Indian Ocean tuna fisheries. Nevertheless, there has been some monitoring of bycatch in purse seine (e.g. [9, 10]), gillnet (e.g. [11, 12]) and longline fisheries (e.g. [13, 14]). Bycatch in pole-and-line fisheries has been even less studied. This is largely because pole-and-line fisheries are widely considered to have little bycatch, with particularly low levels of discards [15, 16], as a result of which there has been little incentive for monitoring.

Within the Indian Ocean the major pole-and-line fishery is that of the Maldives. This fishery has been in existence for at least 800 years [17], and currently accounts for nearly a quarter of the global tuna catch by pole-and-line [18, 19]. In the Maldives, 85,221 t of tuna were landed by pole-and-line in 2015, down from a peak landing of 166,000 t in 2006 [20]. The Maldives boasts the highest per capita fish consumption in the world [21], with over half of all fish caught consumed locally rather than exported (53%, or 64,000 t in 2012) [22]. Much of the social fabric of the country, especially in the smaller, more remote islands, is still closely linked to tuna fishing, with the pole-and-line tuna fishery employing 8,846 fishermen directly in 2014 [23].

As with other pole-and-line tuna fisheries, Maldivian bycatch is considered to be minor, but has not been properly monitored. The relatively few reports all describe the same major bycatch species in small quantities. Rainbow runners (*Elagatis bipinnulata*) were described as often taken as bycatch in the pole-and-line fishery [24, 25]. Species other than tunas (primarily rainbow runner, silky shark [*Carcharhinus falciformis*] and dolphinfish [*Coryphaena hippurus*]) probably contributed ‘less than 5% of the total catch’ from the pole-and-line fishery [26]. The catch of ‘non-target species (mostly dolphinfish and rainbow runners) is negligible and is not reported, nevertheless these fish are landed and consumed’ [25]. Silky sharks were frequently taken as bycatch by pole-and-line vessels, although most of the sharks were actually caught by handline and by hand [27]. Silky shark was considered the most important bycatch species [27], but following a ban on shark fishing in the Maldives in 2010, silky shark catch (and consequently total bycatch) by pole-and-line vessels has decreased [28]. Minor quantities of other species have been noted, including ocean triggerfish (*Canthidermis maculatus*), tripletail (*Lobotes surinamensis*) and oceanic whitetip shark (*Carcharhinus longimanus*) [26].

With the exception of these essentially anecdotal reports, bycatch in the Maldivian pole-and-line tuna fishery is undocumented and unquantified. However, there is a pressing need for more detailed information on bycatch from all tuna fisheries. This is required both to monitor the status of vulnerable bycatch species [29] and to meet sustainability criteria [30]. More generally, under the UN Convention on the Law of the Sea of 1982 (which underpinned the original Agreement for the Establishment of the Indian Ocean Tuna Commission [IOTC]), there is a specific requirement (Article 119, Conservation of the living resources of the high seas) to ‘take into consideration the effects on species associated with or dependent upon harvested species with a view to maintaining or restoring populations of such associated or dependent species above levels at which their reproduction may become seriously threatened’ [31]. Monitoring and assessing bycatch can contribute towards responsible stewardship, sustainable utilization and the development of precautionary, ecosystem-based fisheries management, which is a declared goal of the IOTC.

This study documents the first dedicated survey of bycatch in a pole-and-line tuna fishery in the Indian Ocean. The aim of the survey was to observe 100 day trips of the Maldivian pole-and-line tuna fishery in order to quantify bycatch and discards, to identify major factors influencing bycatch rates, and to highlight areas of concern.

## Methods

### Study site and fishery

**Fig 1:**
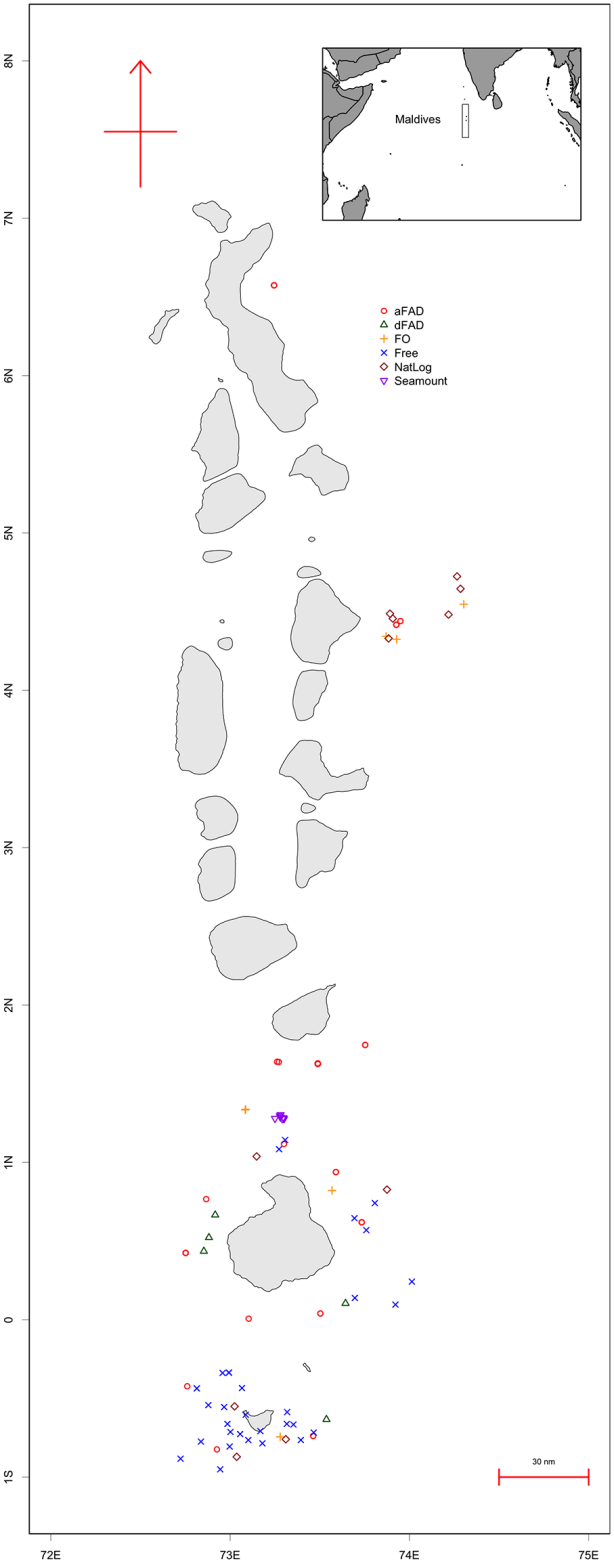
Map of Maldives.

Permission to conduct this research within the Maldives was granted by the Marine Research Centre, Republic of Maldives; no specific permits were required. The Republic of Maldives is an archipelagic atoll chain straddling the equator in the central Indian Ocean, running north-south along 73°E from about 7°N to 1°S (Fig 1). There are some 1,200 islands, grouped across 26 natural atolls. Some 185 of these islands are inhabited.

Pole-and-line fishing is carried out using traditional fishing boats known as *mas dhonis*. There are currently about 1,000 such vessels, which are typically about 18–30 m long. While the fishery remains fundamentally the same as it has been for centuries, mechanization and technological improvements and mechanization have dramatically increased efficiency in recent decades.

The livebait pole-and-line fishery consists of two distinct components: catching baitfish and catching tuna. In the Maldives these are invariably conducted from the same vessel, typically within the same fishing trip. Baitfish are usually caught inside the atolls using simple lift-nets deployed from the boat and subsequently kept alive in the vessel. A variety of small fishes are used as livebait, the most important being the silver sprat, *Spratelloides gracilis* (see S3 Table). The Maldivian baitfishery has been relatively well-studied [32–36] and this component of the livebait pole-and-line fishery is not considered further here.

Tuna fishing occurs offshore, in the open ocean, typically within about 12–70 nautical miles (22–130 km) of the atolls [37]. Fishermen locate schools of tuna most frequently by the presence of seabirds or another fishing boat, or by association with a floating object. Floating objects include natural logs, drifting Fish Aggregating Devices (dFADs, originating from the western Indian Ocean purse seine fishery) and Maldivian anchored Fish Aggregating Devices (aFADs). The Maldivian Ministry of Fisheries and Agriculture maintains a network of some 50 aFADs deployed throughout the country, mostly within about 12–15 nautical miles (22–28 km) of the atolls [38]. Approximately 50% of the Maldivian pole-and-line tuna catch is currently taken from these aFADs [39], up from an estimated 44% in the early 1990s [40].

When a school of tuna is located, water is sprayed on the surface of the sea and livebait are thrown into the water, to encourage a feeding frenzy. Several (5–15) fishermen stand at the stern of the vessel and use non-baited barbless hooks with lures to catch the tuna and flick them on board. Fish are stored on ice, and boats typically return to shore to sell their catch the same day.

### Bycatch monitoring

In this study we considered all tuna landings to be ‘catch’, with everything else being ‘bycatch’, following the definition of the Indian Ocean Tuna Commission [41]. ‘Bycatch’ is subdivided here into ‘byproduct’ (non-tuna species that are retained for sale or consumption), ‘discards’ (any catch that is discarded dead, including damaged tunas) and ‘release’ (any catch that is released alive). However, we summarize our results in sufficient detail that they can be re-worked under different definitions.

One hundred and six fishing days were observed between August 2014 and November 2015 (S1 Table) by experienced fishery observers. In most cases (n = 89) there were two observers, on 17 days three observers and a single observer on just one trip. The trips were planned to cover both monsoon seasons, and all regions of the Maldives, but with most effort in the south where pole-and-line fishing is now concentrated.

On site, vessels were chosen opportunistically; vessels ranged from 16–35 m in length, with 8–23 crew. Most fishing trips started in the late evening, included night baitfishing, a full day searching and fishing for tuna offshore, and a return to shore in the early evening. On one occasion the fishing boat stayed out at sea overnight, tuna fishing on two successive days (thus there were 105 fishing trips, and 106 observed days). There were 86 trips in which tuna fishing was conducted, on 87 days. Most trips in which fishing was not conducted consisted of either trips with insufficient bait catch or days in which schools of tuna could not be located. Mean trip duration was 22h 08min (+/- 6h 45min). During the 106 days, baitfishing was carried out on 86 days, with 99 baitfishing events observed (on the other 20 occasions livebait was still available from the previous trip). A troll line was sometimes towed behind the fishing vessel, while searching for tuna. Three skipjack and one brown lesser noddy (*Anous tenuirostris)* were caught by troll, but are included with the other pole-and-line catch and bycatch in the results tables.

During tuna fishing, detailed records of fishing activities and catch were maintained. Effort data included vessel details, number of fishermen/hooks, amount and type of bait used. Position, school association, sighting method and duration were recorded for each fishing event. Fishing events were defined as periods of active fishing that were separated by more than 10 minutes. School association was recorded as one of six categories: anchored FAD (aFAD), drifting FAD (dFAD), natural log, floating man-made object, seamount, or free school. Schools were defined as associated if the start of fishing was within 1 nautical mile (1.85 km) of any FAD or other floating object, or within 5 nautical miles (9 km) of a seamount following [9].

Because of safety concerns, sampling of the catch and bycatch could not be carried out during active fishing. Sampling was carried out during fishing events if there were brief stoppages in active fishing while the vessel steamed to reposition on the tuna school. Otherwise, sampling was carried out at the end of each fishing event.

For tuna catch, it was not possible to measure or weigh all skipjack and yellowfin caught, as fishermen were keen to put the catch on ice as quickly as possible. One hundred tunas were identified to species and measured (fork length) per fishing event. Also, for each fishing event, the total weight of tuna caught was estimated, and the proportion of skipjack and yellowfin was calculated based on number and average weight of 100 fish. For bigeye tuna (which were not separated from yellowfin tuna at sale), numbers were estimated by close inspection of the external appearance of a subset of up to 50 *Thunnus* per fishing event. Numbers of minor tuna species (frigate tuna [*Auxis thazard*] and kawakawa [*Euthynnus affinis*]) were counted on board immediately after capture; subsamples were measured and weighed. The total weights of major species (skipjack and yellowfin) were recorded when catch was weighed at sale.

For bycatch species, every individual caught was identified to the lowest taxon possible, counted, and its fate recorded. A subsample of each species was measured and weighed. The estimated size and condition of every individual discarded or released was recorded.

Qualitative notes describing activities were recorded daily, and a written report produced at the end of each multi-day sampling trip. A detailed description of the observer sampling protocol is available in [42].

### Data analysis

As it was not possible to measure or weigh all catch and bycatch, some weights were estimated. For the main tuna species (skipjack and yellowfin), almost all the catch was sold at the end of each day’s fishing and was weighed by the buyer at the time of sale. In some cases, skipjack and yellowfin were weighed separately. In other cases, when skipjack and yellowfin were weighed together, the total weight was apportioned between the species according to the recorded proportions in the catch and their average weights. Average weights were estimated from the lengths measured, using established length-weight conversion factors (S2 Table). On days when there was more than one fishing event, total catch was then further apportioned between fishing events based on catch estimates made on board. Bigeye catch was estimated as a proportion of the *Thunnus* catch (i.e. what had been sold as yellowfin tuna), based on the sampling carried out that day.

Occasionally, bycatch individuals could not be weighed (e.g. when released alive by fishermen); in these cases it was sometimes possible to measure lengths, but more frequently lengths were estimated. Species specific length–weight relationships (S2 Table) were then applied to convert into weights. Two-tailed Mann-Whitney U tests were performed on bycatch rates for each fishing event.

## Results

**Fig 2:**
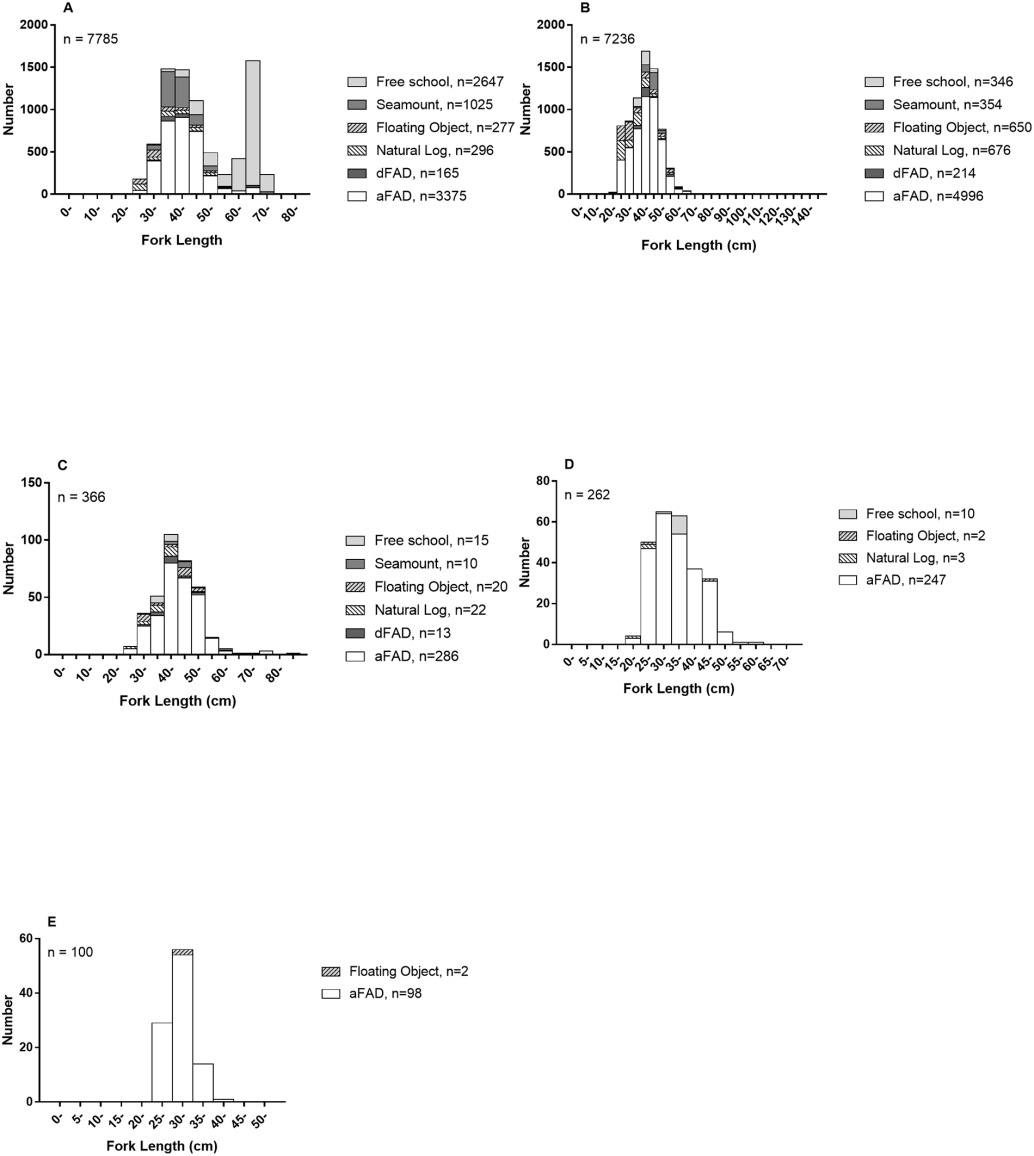
Length frequencies of Maldivian pole-and-line tuna. Species caught include skipjack (A), yellowfin (B), bigeye (C), kawakawa (D), and frigate (E).

Observers spent 106 days at sea (104 one-day trips, and one two-day trip). Pole-and-line fishing was carried out on 87 of these days, during which there were 161 separate fishing events (Fig 1, Table 1). A total of 146,593 kg of tuna was caught. The main tuna species taken were skipjack (72.3% of the tuna catch by weight) and yellowfin (25.0%), with bigeye, kawakawa, and frigate tuna together making up the balance (2.7%) (Table 2). Tuna school composition varied by school association, with a greater proportion of skipjack at free schools than associated schools (U = 307, p<0.0001). All tunas, even small ones, were retained for sale or home consumption, and the only tunas that were discarded were one skipjack and one yellowfin which had been depredated by sharks (Table 3 and S3 Table). 15,749 tunas were measured (S4 and S5 Tables), and length frequencies are summarised in Fig 2. Tuna catches by region are summarised in S6 Table.

**Table 1:** Summary of fishing effort and tuna catch by school association. FE = fishing event.

School association	Fishing Events (n)	% Fishing Events	Total tuna (kg)	% tuna caught	Catch per FE (kg)
**Free schools**	37	23.0%	66,577.4	45.4%	1799.4
aFAD	89	55.3%	48,148.8	32.8%	541.0
dFAD	5	3.1%	2,665.0	1.8%	533.0
natural log	11	6.8%	4,033.2	2.8%	366.7
other floating	8	5.0%	9,860.1	6.7%	1232.5
All floating objects	113	70.2%	64,707.1	44.1%	572.6
Seamount	11	6.8%	15,308.1	10.4%	1391.6
**All Associated**	124	77.0%	80,015.3	54.6%	645.3
**Total**	161	100.0%	146,592.7	100.0%	910.5

**Table 2:** Maldivian pole-and-line tuna catch by school association.

School Association	Catch (kg)	Skipjack	Yellowfin	Bigeye	Kawakawa	Frigate
**Free schools**	66,577.4	98.1%	1.8%	0.1%	0.02%	0.0%
aFAD	48,148.8	49.1%	44.0%	3.7%	3.0%	0.2%
dFAD	2,665.0	58.7%	39.5%	1.9%	0.0%	0.0%
natural log	4,033.2	43.8%	53.4%	2.7%	0.03%	0.0%
other floating	9,860.1	44.2%	53.7%	2.1%	0.02%	0.0%
All floating objects	64,707.1	48.4%	45.9%	3.3%	2.2%	0.1%
Seamount	15,308.1	61.4%	37.5%	1.1%	0.0%	0.0%
**All associated**	80,015.3	50.9%	44.3%	2.9%	1.8%	0.1%
**Total**	146,592.7	72.3%	25.0%	1.6%	1.0%	0.1%

**Table 3:** Maldivian pole-and-line bycatch numbers and sizes, by species.

Species	No. caught	No. measured	Mean length (cm)	No. weighed	Mean weight (kg)	Total weight (kg)
Rainbow runner	265	148	53.4	265	2.12	561.7
Dolphinfish	157	111	60.9	150	2.04	319.9
Silky shark	9	2	73.2	2	2.12	19.1
Round scad	56	27	29.2	53	0.50	28.2
Garfish	12	11	76.5	12	0.95	11.4
Oceanic trigger	7	7	31.0	6	0.71	5.0
Jacks / trevallies	13	13	19.5	13	0.14	1.9
Lesser noddy	1	0	-	0	0.10	0.1
Brown noddy	2	0	-	0	0.20	0.4
Skipjack tuna	1	0	-	0	1.5	1.5
Yellowfin tuna	1	0	-	0	1.5	1.5
Total	524	319	-	501	-	950.57

**Fig 3:**
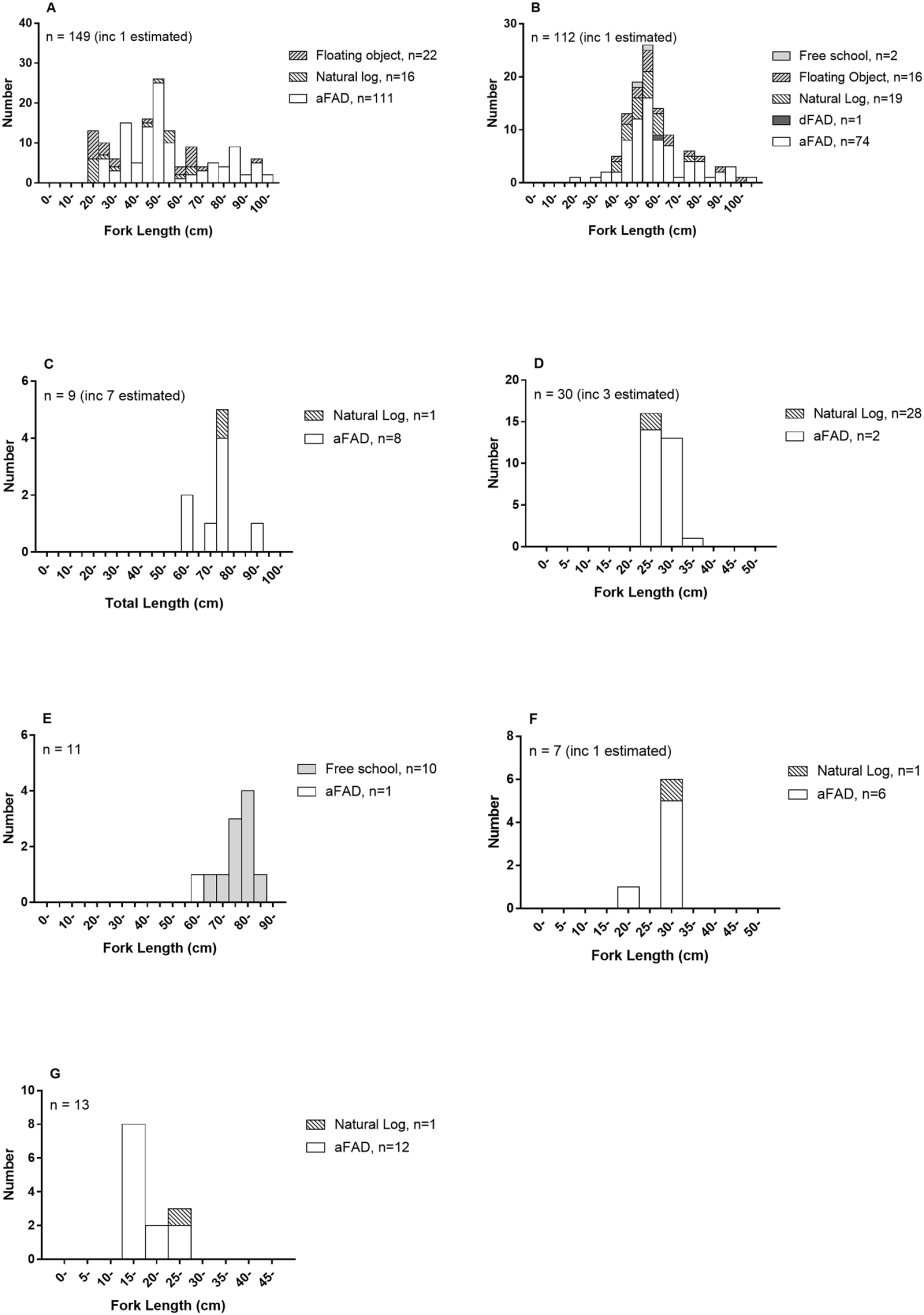
Length frequency of pole-and-line bycatch. Species include rainbow runner (A), dolphinfish (B), silky shark (C), round scad (D), garfish (E), oceanic triggerfish (F), and jack/trevally (G).

Nine bycatch species (or species groups) were taken (Table 3 and S3 Table), and length frequencies are summarized in Fig 3. The most common bycatch species were rainbow runner and dolphinfish. Note that all dolphinfish were recorded as one species (they are believed to have been all common dolphinfish, *C*. *hippurus*, but it is possible that some pompano dolphinfish, *C*. *equiselis*, were caught). Note also that most trevally were only identified to genus, and are therefore grouped together. Nine individual sharks (all silky shark) and three individual seabirds (one lesser noddy, *Anous tenuirostris* and two brown noddies, *A*. *stolidus*) were caught. Of the sharks and seabirds, all except two sharks (which died) were released alive, without apparent injury. Note that the lesser noddy (plus three skipjack) was caught by troll line, towed behind the vessel. Although these catches were not taken strictly by pole-and-line, all these animals were taken during the course of pole-and-line fishing trips, and for simplicity are summarised in all the results tables as pole-and-line bycatch. No marine mammals or turtles were caught during baitfishing or pole-and-line operations.

There was a total of 951 kg of bycatch (all non-tuna catches plus tuna discards) (Table 3). That was 0.65% of the tuna landings, or 0.64% of all catch and bycatch. Bycatch composition was closely related to school association (Table 4). Bycatch was taken in just 5.4% of all free school fishing events, but in 52% of all associated school fishing events. Furthermore, bycatch from free schools was only 0.02% of the tuna catch by weight, whereas from associated schools it was 1.21%, and the bycatch rate was significantly higher at all associated schools (U = 995.5, p <0.0001). There were not enough samples to assess differences between different types of associated schools. Note that 55% of the fishing events (and 33% of catch) occurred at aFADs (Table 1). The quantities of major bycatch species taken by region (North v South) are summarized in Table 5. Bycatch rates, not accounting for school type, were significantly higher in the north than in the south (U = 1663, p = 0.0001).

**Table 4:** Maldivian pole-and-line tuna and bycatch, by school association.

School Association	Catch (kg)	Bycatch (kg)	Bycatch (%)	Bycatch (n)
**Free schools**	66,577.4	13.9	0.02%	14
aFAD	48,148.8	720.4	1.50%	399
dFAD	2,665.0	2.0	0.08%	1
natural log	4,033.2	57.5	1.43%	44
other floating	9,860.1	148.8	1.51%	62
All floating objects	64,707.10	928.7	1.44%	506
Seamount	15,308.1	7.9	0.05%	4
**All associated**	80,015.3	936.6	1.17%	510
**Total**	146,592.7	950.5^a^	0.65%	524

^a^Does not include 0.1 kg bycatch from trolling between pole-and-line fishing events.

**Table 5:** Bycatch by major species in the north and south of the Maldives. Weighted ratios between regions are based on 34d fishing in the north and 72d in the south.

	Rainbow runner	Dolphinfish	Round scad	All bycatch
Numbers				
North	214	25	51	300
South	51	132	5	224
N:S ratio	4.2	0.2	10.2	1.3
N:S weighted	8.9	0.4	21.6	2.8
Weight				
North	438.9 kg	60.3 kg	26.8 kg	537.3 kg
South	122.8 kg	259.6 kg	1.4 kg	413.2 kg
N:S ratio	3.6	0.2	19.1	1.3
N:S weighted	7.6	0.5	40.5	2.8

Ninety-five percent (95%) by weight of the bycatch was retained for sale or consumption, i.e. was byproduct (Table 6). Live releases amounted to 0.01% of the tuna catch or 2% of bycatch, while dead discards were 0.02% of the tuna catch (3% of bycatch). The fate of each bycatch species is summarized in Tables 7 and 8. Discards amounted to 32 kg, equivalent to 22 kg per 100 t of tuna catch, or 0.02% of tuna catch. Over half of the discards (by both numbers and weight) was of dolphinfish. The remainder was of several species, the most important being silky sharks and oceanic triggerfish. Also included in the discards were two partial tunas that had been depredated by sharks.

**Table 6:** Fate of bycatch and discards taken by Maldivian pole-and-line (during 106 observed fishing days with 146,593 kg of tuna caught).

	Bycatch (n)	Bycatch (kg)	Bycatch (%)	% of tuna catch
Byproduct (all non-tunas retained)	489	902.7	95.0%	0.62%
Byproduct (retained for sale)	308	551.8	58.1%	0.38%
Byproduct (retained for consumption)	181	350.9	36.9%	0.24%
Discarded and released	35	47.9	5.0%	0.03%
Discarded (dead or in poor condition)	25	31.8	3.3%	0.02%
Released (alive)	10	16.1	1.7%	0.01%
Bycatch (all non-tunas and discards)	524	950.7	100%	0.65%

**Table 7:** Fate of bycatch and discards, by species (in numbers).

	Sold	Consumed	Discarded	Released	Total
Rainbow runner	212	53	-	-	265
Dolphinfish	48	96	13	-	157
Silky shark	-	-	2	7	9
Round scad	48	8	-	-	56
Garfish	-	12	-	-	12
Oceanic triggerfish	-	-	7	-	7
Trevally/Jacks	-	12	1	-	13
Noddies	-	-	-	3	3
Yellowfin tuna	-	-	1	-	1
Skipjack tuna	-	-	1	-	1
Total	308	181	25	10	524

**Table 8:** Fate of bycatch and discards, by species (by weight, in kg).

	Sold (kg)	Consumed (kg)	Discarded (kg)	Released (kg)	Total (kg)
Rainbow runner	436.0	125.7	-	-	561.7
Dolphinfish	90.4	209.5	20.0	-	319.9
Silky shark	-	-	3.5	15.6	19.1
Round scad	25.5	2.7	-	-	28.2
Garfish	-	11.4	-	-	11.4
Oceanic triggerfish	-	-	5.0	-	5.0
Trevally/Jacks	-	1.6	0.3	-	1.9
Noddies	-	-	-	0.5	0.5
Yellowfin tuna	-	-	1.5	-	1.5
Skipjack tuna	-	-	1.5	-	1.5
Total	551.8	350.9	31.8	16.1	950.6

## Discussion

The bycatch rate recorded here (0.65% of the tuna catch) is very similar to that recorded from western Pacific pole-and-line tuna fisheries. In a review of the limited and scattered literature, average bycatch was estimated at 0.8% of the total catch for western Pacific pole-and-line skipjack fisheries, with rainbow runner and dolphinfish being the most frequently taken bycatch species [43]. Observer data from the Solomon Islands gave an estimated bycatch of 0.6% of the pole-and-line tuna catch, with rainbow runner and dolphinfish again being the most frequently taken bycatch species [44]. In Indonesia, bycatch was estimated at 1% in small-scale tuna pole-and-line fisheries [4]. In a global review, a mean discard rate of 0.4% from tuna pole-and-line fisheries was estimated [15].

By most standards the bycatch rate reported here, and in other tuna pole-and-line fisheries, is low. The discard rate reported here is particularly low. While extrapolation of such a small sample to the entire fishery has very low confidence, it gives a first indication of the likely scale of these bycatch categories. The ratio estimator method indicated, based on the 2015 total catch (85,221 t), 27.9 t of discards, of which 18.5 t were dead discards, and 552.6 t of bycatch for the entire Maldivian pole-and-line fishery. Of this, 524.8 t would be utilized as byproduct. It is stressed that these estimates are very crude and unlikely to be robust, as the sample size (just 106 days fishing) is relatively small, and there are insufficient data to warrant stratification by school type, region or season. However, the issue of waste, which is a major issue in many fisheries, is shown to be of minor concern for the case of the Maldives pole-and-line fishery. Nevertheless, there is scope for improvement, and options for utilization of the small quantities of fish currently discarded should be explored.

Among the species (and species groups) taken as bycatch, almost all (with the exception of noddies) have been recorded before from tuna pole-and-line fisheries in both the Maldives (see Introduction), and the western Pacific [43, 44], and all are common and widespread. None are considered endangered [45].

The one species for which there is some concern is the silky shark, which has been assessed as Near Threatened [45] and is known to have declined in abundance in Maldivian waters and in the wider Indian Ocean [6, 46]. A precautionary approach to its management has been recommended by the IOTC Scientific Committee [47]. All forms of shark fishing were banned in the Maldives from March 2010 [28]. In common with most other sharks, the silky shark’s life history characteristics (longevity, delayed maturation, low fecundity) make it particularly vulnerable to over-fishing. There is scope for further education of fishermen on the protected status of sharks, and on techniques for reducing bycatch and facilitating safe release if hooked. The Maldives initiated a proposal to CITES (the Convention on International Trade in Endangered Species of Wild Fauna and Flora) CoP17 for silky shark to be listed in its Appendix II, which passed in 2016.

During this study, three seabirds (one lesser noddy and two brown noddies) were caught, but all were released and flew away apparently unharmed. Both of these bird species are common in the Maldives and the wider region, and the conservation status of both has been assessed as Least Concern [45]. Maldivian fishermen frequently use the presence of seabirds to locate tuna schools [48–51]. The importance of seabirds is widely recognized by Maldivian fishermen [50, 51], and it was mainly for this reason that these species, and several other seabirds, are protected under Maldivian law (Directive 10-C/99/24 of 11 July 1999 under the Environment Protection and Preservation Act 4/93) [52]. As a result of this legal protection, the capture of seabirds by Maldivian fishermen, which was widespread, is now rare ([49, 50, 53], authors' pers. obs.). Nevertheless, the hooking of three seabirds in just 106 days’ fishing does raise some questions. All three noddies were apparently uninjured, and flew away strongly on release, although the possibility of sublethal impacts, or post-release mortality, cannot be entirely dismissed. It would be inappropriate to multiply up from such a small sample, so the total number of seabirds being hooked in this fishery is unknown. Nevertheless this is an issue that deserves further study. At the same time there is scope for futher education of fishermen on the importance of seabirds to the fishery, their protected status, and techniques to avoid capture and to facilite safe release if hooked.

### Variation in bycatch rates

The results of this study demonstrate that there is considerable variability in bycatch rates. The most important factor influencing bycatch rate was tuna school association. Free schools were typically composed almost entirely of large skipjack, and yielded little bycatch. In contrast, associated schools contained mostly smaller-sized skipjack together with juvenile yellowfin and bigeye tunas, and yielded much more bycatch. Similar differences in tuna composition and bycatch rates between free schools and FAD schools are known from the purse seine tuna fisheries (e.g. [9, 54]), which also show variation by ocean (region) and retention or discard of small tunas. Indeed, purse seine bycatch ranged from a low of 0.9% for seamount-associated schools in the Indian Ocean [9] to 15.0% for FAD-associated schools in the Atlantic [55]. For the Maldivian pole-and-line fishery there may be differences between different types of associated schools, but the quantities of bycatch taken from the different school types during this study are too small to make meaningful comparisons.

Within the Maldives there are clear regional differences in bycatch composition. Bycatch rates in the north of Maldives (north of 2°N) were significantly higher (up to three times greater) than in the south (Table 5). There were also differences in species composition. Bycatch from the north included more rainbow runners and round scad, while dolphinfish dominated in the south. Among tunas, bigeye was relatively more abundant in the south of Maldives, while kawakawa and frigate tuna were relatively more abundant in the north. Differences in relative abundance of different pelagic fish species between these regions have been attributed to differences in oceanographic conditions [56].

It should be noted that Maldivian pole-and-line fishermen can clearly see what they are catching, and will not continue catching species which they cannot sell or consume. During this survey it was apparent that there was greater demand for bycatch species in the north of Maldives than in the south. This was partly because the main southern fish buyers would only buy skipjack, yellowfin and bigeye, while northern buyers would buy other species as well. Also, many fishermen in the north have access to Malé fish market (by far the largest in the country), where they could sell many species of fish. These factors may have encouraged northern fishermen to take more bycatch.

It is likely that there are also seasonal differences in bycatch. However, these are not apparent in our data, in part perhaps because of the relatively small sample sizes.

### The issue of bait

In this study, as in most similar studies, the livebait component of the pole-and-line fishery was not considered bycatch due to different target species, different gears, different locations, and different management issues. For these reasons the catch of livebait is usually excluded from the definition of bycatch [13, 14, 15, 44]. One exception to this standard practice was provided by [25], wherein livebait was included in bycatch estimates for the Maldivian pole-and-line fishery and, based on published information from MRC, noted bycatch at 11.6% of the tuna catch. For comparison with [25], during this study it was estimated that total livebait catch was 13.0 t. That was equivalent to 8.9% of the tuna catch, or 11.3 kg of tuna per 1 kg of livebait caught. This is comparable to, but slightly more efficient than, catch rates estimated by previous Maldives studies (summarised in [36]), which reported catches within the range of 7.4–10.0 kg of tuna per 1 kg of livebait used, or 10.0–13.5% of tuna catch.

### Tuna catches

While this report deals specifically with bycatch, some information on the tuna catch is relevant to interpreting the bycatch data.

Different school types showed markedly different tuna species composition and different sizes of skipjack tunas (Table 2 and Fig 2A). Skipjack made up nearly all (98%) the tuna catch from free schools but only half (51%) from associated schools. Furthermore, the skipjack caught from free schools were mostly uniformly large-sized, whereas smaller skipjack predominated in associated-school catches (Fig 2A and S5 Table). Across the entire study, skipjack lengths showed a bimodal distribution (Fig 2A); this is a well-known feature of Maldivian skipjack landings [57, 58]. Also, across the entire study, skipjack contributed 72.3% to the total tuna catch, which is close to the long-term estimated average for the national fishery of 75% [59].

The yellowfin tuna taken by pole-and-line were all juveniles, with an average fork length of roughly 40 cm in both free schools and associated schools (Fig 2B, S5 Table). This agrees with previous findings [60].

Juvenile bigeye tuna makes up a small proportion of the Maldivian pole-and-line catch (Table 2), but these fish are not always easy to distinguish from juvenile yellowfin tuna. Indeed, bigeye catches were generally not separated from yellowfin catches in official Maldivian catch statistics until 2014, when bigeye catches began being recorded separately in the logbooks. In this study, the proportion of bigeye in the *Thunnus* component of the pole-and-line catch was estimated at 6.1%, but with a higher proportion in the south (7.3%) than the north (2.7%) (S7 Table). This agrees with previous studies [61–63], which all found a higher proportion of bigeye in the south of Maldives than in the north and centre (S7 Table). In neighbouring Sri Lanka, inspection of ‘small yellowfin’ landings at Beruwela (6°29’N, the same latitude as Shaviyani Atoll in the north of Maldives) found 0.7% bigeye [64]. The cause of this latitudinal variation in bigeye tuna abundance is unknown, but may be related to a known deep oxygen minimum zone [65, 66] that could affect deep-swimming species such as bigeye.

### Comparison with other fisheries

In comparison to other fisheries, a bycatch rate of 0.5%-1% is very low. For example, there are estimates of global average bycatch from shrimp trawl of 62%, tuna longline of 28%, and tuna purse seine at 5% [15]. Worldwide, total pole-and-line fishing discards were estimated at 3,121 t, compared with 144,152 t for tuna purse seine in 2005 [15].

Within the Indian Ocean, in addition to pole-and-line, the other major tuna fishing gears are gillnet, purse seine and longline. In 2015, reported catches for tuna and tuna-like species were 534,000 t for gillnet (33% of the total), 415,000 t for purse seine (26%) and 260,000 t for longline (16%), compared to 102,000 t for pole-and-line (6%) (catch data from www.iotc.org, accessed January 2017).

Gillnet fisheries are not only the largest tuna fisheries in the Indian Ocean but are also believed to have the highest levels of bycatch. However, they have been poorly documented. Most of the bycatch sampling to date has been of landings, not catches, so discards are unknown. However, recent research in Pakistan, which incorporated observer data, reported bycatch amounting roughly 40% of tuna landings [67, 68]. If that were representative of all Indian Ocean tuna gillnet fisheries, then it would suggest bycatch in excess of 210,000 t in 2015. It should be noted, however, that a large part of the bycatch is of other edible finfish and of sharks, most of which are sold as byproduct.

The tuna purse seine fishery in the Indian Ocean is dominated by French and Spanish fleets. For these fleets, bycatch was sampled during 2003–2009 by onboard observers, and estimated at 11,590 t per year, which was 4.7% of tuna landings [69]. This likely underestimated total bycatch, since marine mammals and whale sharks were caught but excluded from the analysis, and rare catches of large numbers of some bycatch species were under-represented in the observer samples [69]. In addition, the proportion of dFAD-sets by tuna purse seiners has been increasing in recent years [70], which has likely resulted in the proportion of bycatch increasing. Nevertheless, if 4.7% were representative of all Indian Ocean tuna purse seine fisheries, then it would suggest bycatch of over 20,000 t in 2015.

Longline tuna fisheries cover all tropical and temperate waters of the Indian Ocean. Although the basic longline technique is fairly standard, there is much variation in gear materials and configuration, in setting depth and between ecological regions. Thus, there is considerable variation in tuna catch and bycatch, and bycatch rates can be particularly high in some tuna longline fisheries. For example, seabird bycatch has been unsustainably high in the southern Indian Ocean (e.g. [5, 71]), while shark bycatch can be especially high in tropical waters (e.g. [72, 73]). With such variability, it is difficult to make a robust estimate of total bycatch. However, a large study of bycatch on Taiwanese tuna longliners which covered much of the Indian Ocean found that bycatch and discards comprised 35.8% of total catch [13]. If this were representative of all Indian Ocean tuna longline fisheries, then it would suggest bycatch of the order of 145,000 t in 2015.

While there is much uncertainty in these bycatch estimates, it is clear that other tuna fisheries, particularly gillnet and longline fisheries, have much higher bycatches than the tuna pole-and-line fishery. And for all of these other fisheries, there is also the problem of ghost fishing gear: lost or discarded pieces of fishing gear which may continue to catch a wide variety of species for months or years [74, 75]. Within the Indian Ocean, ghost gillnets and purse seine FADs have been implicated in the mortality of particularly large numbers of silky sharks and olive ridley turtles, *Lepidochelys olivacea* [75, 76]. Such mortalities should be considered as part of the bycatch of the gillnet and purse seine fisheries. There is very little gear loss and effectively no ghost fishing from the pole-and-line fishery.

One of the more striking aspects of the pole-and-line bycatch recorded here is not what was included in the bycatch, but what was not present. While it is not surprising that whales, dolphins or turtles were not caught, the almost complete absence of discarded tuna is of note. In this study, only one skipjack and one yellowfin tuna were discarded, both depredated by sharks. Also during the course of this study, in interviews with 10 fishermen from 10 different boats, fishermen reported that they never discarded tunas because of size or damage; any tuna that could not be sold to processing plants would be retained for sale on local islands or for household consumption. In contrast, discards of small tunas have been a regular feature of western Indian Ocean purse seine fisheries. Thus, for the Soviet/Russian purse seine fishery during 1986–92, small tuna discards were estimated at about 8.3 t per 1000 t of tuna retained [10]. And for the European purse seine fishery, it was reported that tuna discards accounted for 54% of all bycatch [9]. However, IOTC [77] has recently banned discarding of skipjack, yellowfin and bigeye tunas (although not frigate tuna or other species) by purse seiners operating in its area of competence (IOTC Resolution 15/06), a move which should reduce tuna discards.

During this study, much of the non-retained bycatch was released alive, unlike many other fisheries in which most fish arrive on deck in poor condition or already dead (e.g. trawl, gillnet, some purse seine). In pole-and-line fishing, fish are brought on deck one at a time, and the flick-off de-hooking technique has very high survival rates [43, 78]. In the Maldivian pole-and-line fishery, the combination of barbless hooks, flick-off de-hooking and short time on deck is believed to promote similarly high survivorship. In comparison, longline tuna fishing can have high mortality rates of discarded fishes: 38–63% discarded dead for Taiwanese tuna longline fisheries [13], and 59% discarded dead for western-central Pacific longline fisheries [44]. In one study for Indian Ocean purse seine fishery, over 50% of the silky sharks brought on board in purse seine operations and released alive subsequently died, raising the total mortality rate of brailed sharks to 85% [79].

Seabird catches are typically minimal in pole-and-line fisheries, although 3 were caught during the course of this study. A review of Pacific pole-and-line bycatch found no records of seabird captures [43]. In a study of the Brazilian skipjack pole-and-line fishery, no seabird captures were reported, although fishermen did deliberately hit seabirds to scare them away [80]. In contrast, in the southern Indian Ocean, tuna longline fisheries have been a major source of mortality for several species of seabirds, particularly petrels and albatrosses (e.g. [5, 13]).

### FADs & unobserved mortalities

As noted above, the Maldivian pole-and-line fishery makes full use of the network of approximately 50 aFADs deployed around the atolls [81, 82]. This network of aFADs was developed by the Government of Maldives to provide an opportunity for fishermen to locate and catch tuna with a relatively high likelihood of success, and it has worked, with roughly one third to one half of the total tuna catch now coming from aFADs ([39], present study).

Most FADs (both anchored and drifting) are deployed with swathes of netting underneath, which are believed to increase their aggregating power, but can lead to animal entanglement and death. In the western Indian Ocean purse seine fisheries, very high numbers of dFADs are employed: more than 7,600 dFADs were estimated to have been deployed in the Indian Ocean in 2009 [83], and 6,700 active dFADs were deployed by the French and Spanish purse seine fleets in the western Indian Ocean at the end of 2013 [84].

It has been estimated that roughly 480,000 to 960,000 silky sharks may be entangled and die annually in the netting of dFADs deployed by the purse seine fleet, a mortality 5 to 10 times higher than the reported catch [76]. There is also a problem for turtles, particularly olive ridley turtles (*Lepidochelys olivacea*), which frequently become entangled in FAD netting [75, 85]. Comparisons between non-entangling and entangling FADs suggest equal tuna attraction, while reducing tangling mortality of other marine life [86, 87]. IOTC has adopted a resolution to phase out entangling FADs, but progress appears to be slow.

The number of Maldivian aFADs is less than 1% of the number of dFADs deployed by the western Indian Ocean purse seine fisheries. The ecological impacts of these two types of FAD must therefore be very different, especially as aFADs tend to have smaller net panels than dFADs [88]. Nevertheless, there is scope for improvement. Maldivian aFADs are deployed with netting attached underneath, and Maldivian fishermen do sometime add extra lengths of netting to aFADs (pers. obs). While no entanglements were observed during this study, there are infrequent anecdotal reports of entangled turtles. Maldives is moving towards net-free aFADs, as recommended by IOTC (Resolution 15/08).

## Conclusions

Bycatch management for large industrial fisheries (e.g. purse seine) frequently focuses on reducing bycatch, and specifically discards. However, the Maldivian pole-and-line fishery has few discards, since most (95%) of the non-tuna catch is edible and is utilised. Therefore, rather than trying to reduce bycatch rates, management efforts should concentrate on reducing impacts on ETP species [4]. For the Maldivian pole-and-line fishery, interactions with ETP species are already limited, but assessing and mitigating impacts on silky sharks and birds should be a priority. One useful line of research would be electronic tagging of released animals to provide confirmation of survival rates, as has been done in the purse seine fishery [79].

The 106 days fishing observed during this study were sufficient to provide a first estimate of bycatch rates for major bycatch species, as intended. However, it is inevitable that with such a small sample size, some rare interactions with ETP species must have been missed by this study. Continuous and long-term monitoring and/or observer coverage would be required to detect such rare events and to gain a more complete understanding of pole-and-line fishery bycatch issues. However, on a regional scale it might be more useful to increase the monitoring of other fisheries (notably pelagic gillnetting) which have more significant bycatch issues.

It should be noted that during this study, observer effects (the change in fishermen’s behaviour with an observer onboard) were believed to be low. This was due to the fact that the Maldivian pole-and-line fishermen did not perceive that they were in any way doing anything untoward: they had nothing to hide. This attitude might change in the future if legal restrictions were introduced.

In summary, this study confirms previous suggestions that bycatch and discards in the Maldivian pole-and-line tuna fishery are low. Because most of the bycatch is used for human consumption, discards from this fishery are particularly low. Nevertheless, there may be some scope to encourage further utilization of some of the very small quantity of low-value fish that is currently discarded, and to further reduce and mitigate the small number of interactions with sharks and seabirds. There is also a need to ensure that all Maldivian aFADs are non-entangling.

## Supporting information

S1 Table Summary of fishing trips observed for bycatch estimation (September 2014 to November 2015). (DOCX)

S2 Table Weight conversion factors for different species. FL = fork length in cm, TL = total length in cm.
(DOCX)

S3 Table Species caught during this study. (DOCX)

S4 Table Mean lengths and weights of tuna species caught. (DOCX)

S5 Table Fork lengths (cm) of skipjack, yellowfin and bigeye tunas by school association. (DOCX)

S6 Table Tuna catch composition by region. (DOCX)

S7 Table Proportion of bigeye tuna in *Thunnus* catch, by region. (DOCX)
