# Supplementary material for: Bycatch in the Maldivian pole-and-line tuna fishery
Source: PLoS One. 2017 May 24;12(5):e0177391. doi: 10.1371/journal.pone.0177391 (PMC5443503; doi:10.1371/journal.pone.0177391)
Supplement: S1 Table — (DOCX) [file pone.0177391.s001.docx]

**Estimates of bycatch and discards in the Maldives pole-and-line tuna fishery - Supplementary materials**

**S1 Table. Summary of fishing trips observed for bycatch estimation** (September 2014 to November 2015).

| **Major trips** | **Fishing Trips** |  |  |  |  | **Fishing trips** | **Tuna fishing days** | **Fishing days with catch** | **Fishing events** | **No. fishing events by association** | | | | | |  |  |
| --- | --- | --- | --- | --- | --- | --- | --- | --- | --- | --- | --- | --- | --- | --- | --- | --- | --- |
|  |  | **Atoll** | **Island** | **Dates** |  |  |  |  |  | **Free**  **school** | **aFAD** | **dFAD** | **Log** | **Floating object** | **Sea-mount** | **Catch (kg)** | **Bycatch (kg)** |
| 1 | 1-4 | Laamu | Maandhoo | 1.9.14 | 8.9.14 | 4 | 3 | 3 | 6 |  | 5 |  |  |  | 1 | 5,441.4 | 24.0 |
| 2 | 5-11 | Gaafu Dhaalu | Thinadhoo | 13.10.14 | 21.10.14 | 7 | 4 | 4 | 4 |  | 4 |  |  |  |  | 3,358 | 60.4 |
| 3 | 12-13 | Laamu | Maandhoo | 28.10.14 | 11.11.14 | 2 | 1 | 1 | 4 |  | 4 |  |  |  |  | 1,000 | 20.6 |
| 4 | 14-21 | Gaafu Alifu | Kooddoo | 18.12.14 | 30.12.14 | 8 | 7 | 7 | 11 | 2 | 2 |  |  |  | 7 | 13,840 | 6.0 |
| 5 | 22 | Laamu | Gan | 20.1.15 | 25.1.15 | 1 | 0 | 0 | 0 |  |  |  |  |  |  | 0 | 0 |
| 6 | 23-27 | Haa Dhaalu | Kulhudhufushi | 19.1.15 | 28.1.15 | 5 | 5 | 5 | 5 |  | 5 |  |  |  |  | 4,181.3 | 4.9 |
| 7 | 28-32 | Gaafu Dhaalu | Thinadhoo | 15.2.15 | 22.2.15 | 5 | 4 | 3 | 6 | 5 | 1 |  |  |  |  | 16,593.3 | 0.1 |
| 8 | 33-38 | Kaafu | Dhiffushi | 8.3.15 | 17.3.15 | 6 | 5 | 5 | 17 |  | 8 |  | 6 | 3 |  | 2,311.1 | 226.1 |
| 9 | 39-47 | Seenu (Addu) | Hulhudhoo | 29.3.15 | 10.4.15 | 9 | 9 | 9 | 21 | 15 | 2 |  | 2 | 2 |  | 40,287 | 199.7 |
| 10 | 48-55 | Gaafu Alifu | Vilingili | 20.4.15 | 1.5.15 | 8 | 7 | 5 | 15 | 3 | 6 | 3 | 2 | 1 |  | 13,341.1 | 40.1 |
| 11 | 56-59 | Gaafu Dhaalu | Thinadhoo | 14.5.15 | 22.5.15 | 4 | 3 | 3 | 4 |  | 4 |  |  |  |  | 3,850.3 | 2.5 |
| 12 | 60-68 | Seenu (Addu) | Hulhudhoo | 22.5.15 | 5.6.15 | 9 | 6 | 6 | 15 | 8 | 4 | 2 | 1 |  |  | 13,520.8 | 10.7 |
| 13 | 69 | Laamu | Maandhoo | 26.4.15 | 27.4.15 | 1 | 1 | 1 | 3 |  | 1 |  |  | 2 |  | 2,001 | 5.1 |
| 14 | 70-75 | Laamu | Gan | 3.8.15 | 15.8.15 | 6 | 3 | 3 | 7 |  | 7 |  |  |  |  | 835.3 | 10.4 |
| 15 | 76-83 | Kaafu | Dhiffushi | 20.8.15 | 30.8.15 | 8 | 7 | 7 | 7 |  | 7 |  |  |  |  | 4,641.8 | 23.5 |
| 16 | 84-98 | Raa | Alifushi | 3.10.15 | 25.10.15 | 15 | 14 | 14 | 23 | 4 | 19 |  |  |  |  | 8,826.2 | 282.9 |
| 17 | 99-106 | Laamu | Maandhoo | 6.11.15 | 17.11.15 | 8 | 8 | 8 | 13 |  | 10 |  |  |  | 3 | 12,564 | 33.6 |
|  |  |  |  |  |  | 106 | 87 | 84 |  |  |  |  |  |  |  | 146,592.7 | 950.6 |
