# Supplementary material for: Bycatch in the Maldivian pole-and-line tuna fishery
Source: PLoS One. 2017 May 24;12(5):e0177391. doi: 10.1371/journal.pone.0177391 (PMC5443503; doi:10.1371/journal.pone.0177391)
Supplement: S2 Table — FL = fork length in cm, TL = total length in cm. (DOCX) [file pone.0177391.s002.docx]

# Estimates of bycatch and discards in the Maldives pole-and-line tuna fishery - Supplementary materials

**S2 Table. Weight conversion factors for different species**. FL = fork length in cm, TL = total length in cm.

| **Species** | **Conversion** | **Source** |
| --- | --- | --- |
| Skipjack tuna | W = 9.510 x 10^-6^ FL^3.210^ | [[1](#_ENREF_1)] |
| Yellowfin tuna | W = 2.863 x 10^-5^ FL^2.897^ | [[1](#_ENREF_1)] |
| Bigeye tuna | W = 8.681 x 10^-6^ FL^3.228^ | [[1](#_ENREF_1)] |
| Dolphinfish | W = 4.992 x 10^-6^ FL^3.077^ | [[2](#_ENREF_2)] |
| Silky shark | W = 8.174 x 10^-6^ TL^2.914^ | [[2](#_ENREF_2)] |
| Round scad | W = 7.8 x 10^-3^ FL^3.14^ | [[3](#_ENREF_3)] |
| Oceanic triggerfish | W = 2.344 x 10^-2^ FL^2.96^ | [[4](#_ENREF_4)] |
| Lesser noddy | 0.1 kg per bird | [[5](#_ENREF_5)] |
| Brown noddy | 0.2 kg per bird | [[5](#_ENREF_5)] |

**References**

1. Anderson R, Adam M, Nadheeh I. Third Fisheries Project, Tuna Research Component: Final report of tuna length and weight frequency sampling activities, 1994-95. Unpublished report, Marine Research Section, Ministry of Fisheries and Agriculture, Malé 30pp. 1996.

2. Anderson RC, Waheed A. Exploratory fishing for large pelagic species in the Maldives. Bay of Bengal Programme, Madras. BOBP/REP/46: 44pp. 1990.

3. Magnusson J, Magnusson JVV. Survey of demersal fish resources in the waters off Cape Verde Islands in May-June 1984. Report IV. Report: summary of information on species. Icelandic International Development Agency/Marine Research Institute, 1987.

4. Froese R, Pauly D. FishBase: ICLARM; 2015 [updated 10/2015; cited 2015 October 9, 2015]. World Wide Web electronic publication]. Available from: <http://www.fishbase.org/>.

5. Olsen KM, Larsson H. Terns of Europe and North America: A&C Black; 1995.
