# Supplementary material for: Bycatch in the Maldivian pole-and-line tuna fishery
Source: PLoS One. 2017 May 24;12(5):e0177391. doi: 10.1371/journal.pone.0177391 (PMC5443503; doi:10.1371/journal.pone.0177391)
Supplement: S3 Table — (DOCX) [file pone.0177391.s003.docx]

# Estimates of bycatch and discards in the Maldives pole-and-line tuna fishery - Supplementary materials

**S3 Table. Species caught during this study.**

| **English Name** | **Scientific Name** | **Maldivian Name** |
| --- | --- | --- |
| ***Tuna Catch*** |  |  |
| Skipjack tuna | *Katsuwonus pelamis* | Kalhubilamas |
| Yellowfin tuna | *Thunnus albacares* | Kanneli |
| Bigeye tuna | *Thunnus obesus* | Loabodu kanneli |
| Kawakawa / little tuna | *Euthynnus affinis* | Latti |
| Frigate tuna | *Auxis thazard* | Raagondi |
| ***Bycatch*** |  |  |
| Rainbow runner | *Elagatis bipinnulata* | Maaniyamas |
| Dolphinfish / dorado / mahimahi | *Coryphaena hippurus* | Fiyala |
| Silky shark | *Carcharhinus falciformis* | Oivaali (*or* ainu) miyaru |
| Round scad | *Decapterus macarellus* | Rimmas |
| Garfish | Belonidae | Tholi |
| Oceanic triggerfish | *Canthidermis maculatus* | Oivaali rondu |
| Lesser noddy | *Anous tenuirostris* | Kurangi |
| Brown noddy | *Anous stolidus* | Maaranga |
| Jacks / trevallies | Carangidae | Handhi |
| ***Main Baitfish*** |  |  |
| Silver sprat | *Spratelloides gracilis* | Rehi |
| Blue sprat | *Spratelloides delicatulus* | Hondeli |
| Anchovy | Engraulidae | Miyaren |
| Cardinalfish | Apogonidae | Boadhi |
