# Supplementary material for: Bycatch in the Maldivian pole-and-line tuna fishery
Source: PLoS One. 2017 May 24;12(5):e0177391. doi: 10.1371/journal.pone.0177391 (PMC5443503; doi:10.1371/journal.pone.0177391)
Supplement: S4 Table — (DOCX) [file pone.0177391.s004.docx]

# Estimates of bycatch and discards in the Maldives pole-and-line tuna fishery - Supplementary materials

**S4 Table. Mean lengths and weights of tuna species caught.**

| Species | No. measured | Mean length | Mean weight | Total catch |
| --- | --- | --- | --- | --- |
| Skipjack tuna | 7,785 | 46.8 cm | 2.93 kg | 106,034.9 kg |
| Yellowfin tuna | 7,236 | 39.7 cm | 1.55 Kg | 36,636.5 kg |
| Bigeye tuna | 366 | 42.3 cm | 1.90 kg | 2,374.2 kg |
| Kawakawa | 262 | 35.4 cm | 1.13 kg | 1,453.4 kg |
| Frigate tuna | 100 | 31.1 kg | 0.53 Kg | 93.7 kg |
| Total | 15,749 |  |  | 146,592.7 kg |
