# Supplementary material for: Bycatch in the Maldivian pole-and-line tuna fishery
Source: PLoS One. 2017 May 24;12(5):e0177391. doi: 10.1371/journal.pone.0177391 (PMC5443503; doi:10.1371/journal.pone.0177391)
Supplement: S5 Table — (DOCX) [file pone.0177391.s005.docx]

# Estimates of bycatch and discards in the Maldives pole-and-line tuna fishery - Supplementary materials

**S5 Table. Fork lengths (cm) of skipjack, yellowfin and bigeye tunas by school association.**

| School Association | n (SKJ) | Length (cm) ± SE (SKJ) | n (YFT) | Length (cm) ± SE (YFT) | n (BET) | Length (cm) ± SE (BET) |
| --- | --- | --- | --- | --- | --- | --- |
| **Free** | 2,647 | 60.6 ± 0.16 | 346 | 41.7 ± 0.55 | 15 | 39.1 ± 1.78 |
| aFAD | 3,375 | 40.4 ± 0.13 | 4,996 | 40.6 ± 0.12 | 286 | 43.1 ± 0.49 |
| dFAD | 165 | 44.4 ± 0.94 | 214 | 42.4 ± 0.51 | 13 | 40.1 ± 1.38 |
| natural log | 296 | 36.5 ± 0.51 | 676 | 33.6 ± 0.32 | 22 | 37.0 ± 1.47 |
| other floating | 277 | 34.5 ± 0.48 | 650 | 34.8 ± 0.44 | 20 | 39.8 ± 1.77 |
| seamount | 1,025 | 38.9 ± 0.15 | 354 | 43.3 ± 0.25 | 10 | 42.5 ± 0.44 |
| **All Associated** | 5,138 | 39.7 ± 0.11 | 6,890 | 39.6 ± 0.11 | 351 | 45.3 ± 1.78 |
| **Total** | 7,785 | 46.8 ± 0.14 | 7,236 | 39.7 ± 0.11 | 366 | 42.3 ± 0.43 |
