# Supplementary material for: Bycatch in the Maldivian pole-and-line tuna fishery
Source: PLoS One. 2017 May 24;12(5):e0177391. doi: 10.1371/journal.pone.0177391 (PMC5443503; doi:10.1371/journal.pone.0177391)
Supplement: S6 Table — (DOCX) [file pone.0177391.s006.docx]

# Estimates of bycatch and discards in the Maldives pole-and-line tuna fishery - Supplementary materials

**S6 Table. Tuna catch composition by region.**

| Region | Skipjack (kg) | Yellowfin (kg) | Bigeye (kg) | Kawakawa (kg) | Frigate (kg) | Bycatch (kg) |
| --- | --- | --- | --- | --- | --- | --- |
| North | 8,722.7 | 9,695.7 | 266.5 | 1,441.2 | 75.7 | 537.3 |
| South | 97,312.2 | 26,940.8 | 2,107.7 | 12.2 | 18.0 | 413.3 |
| Total | 106,034.9 | 36,636.5 | 2,374.2 | 1,453.4 | 93.7 | 950.6 |
