# Supplementary material for: Bycatch in the Maldivian pole-and-line tuna fishery
Source: PLoS One. 2017 May 24;12(5):e0177391. doi: 10.1371/journal.pone.0177391 (PMC5443503; doi:10.1371/journal.pone.0177391)
Supplement: S7 Table — (DOCX) [file pone.0177391.s007.docx]

# Estimates of bycatch and discards in the Maldives pole-and-line tuna fishery - Supplementary materials

**S7 Table. Proportion of bigeye tuna in *Thunnus* catch, by region.**

| North & Centre | South | Total | Source |
| --- | --- | --- | --- |
| 0.2% | 11.7% | 1.5% | [[1](#_ENREF_1)] |
| 1.3% | 14.7% | 4.6% | [[2](#_ENREF_2)] |
| 4.0% | 21.8% | 9.1% | [[3](#_ENREF_3)] |
| 2.7% | 7.3% | 6.1% | This study |

**References**

1. Anderson RC, Hafiz A. How much bigeye tuna in Maldivian yellowfin catches? IPTP Collective Volume of Working Documents. 1991;6:50-52.

2. Anderson RC. Bigeye tuna (*Thunnus obesus*) in the Maldives. Maldives Marine Research Bulletin. 1996;2:41-54.

3. Adam MS, Jauharee AR, Ahusan M. Notes on Yellowfin / Bigeye Tuna Ratio and Size Distribution in the Maldivian Fishery. Bali, Indonesia: Ministry of Fisheries & Agriculture. Indian Ocean Tuna Commission, 2014 IOTC-2014-WPTT16-26.
